# Correction to “Bioinspired Polyacrylic Acid‐Based Dressing: Wet Adhesive, Self‐Healing, and Multi‐Biofunctional Coacervate Hydrogel Accelerates Wound Healing”

**DOI:** 10.1002/advs.76448

**Published:** 2026-07-03

L. Wang, L. Duan, G. Liu, et al., “Bioinspired Polyacrylic Acid‐Based Dressing: Wet Adhesive, Self‐Healing, and Multi‐Biofunctional Coacervate Hydrogel Accelerates Wound Healing,” *Advanced. Science*. (2023): 10, no. 16 2207352, https://doi.org/10.1002/advs.202207352.

During the figure preparation of the manuscript, two images in **Figure 4F** and two images from Supporting Information in **Figure** **S20** were unintentionally duplicated. Please find the corrected versions of Figures 4 and S20 below:


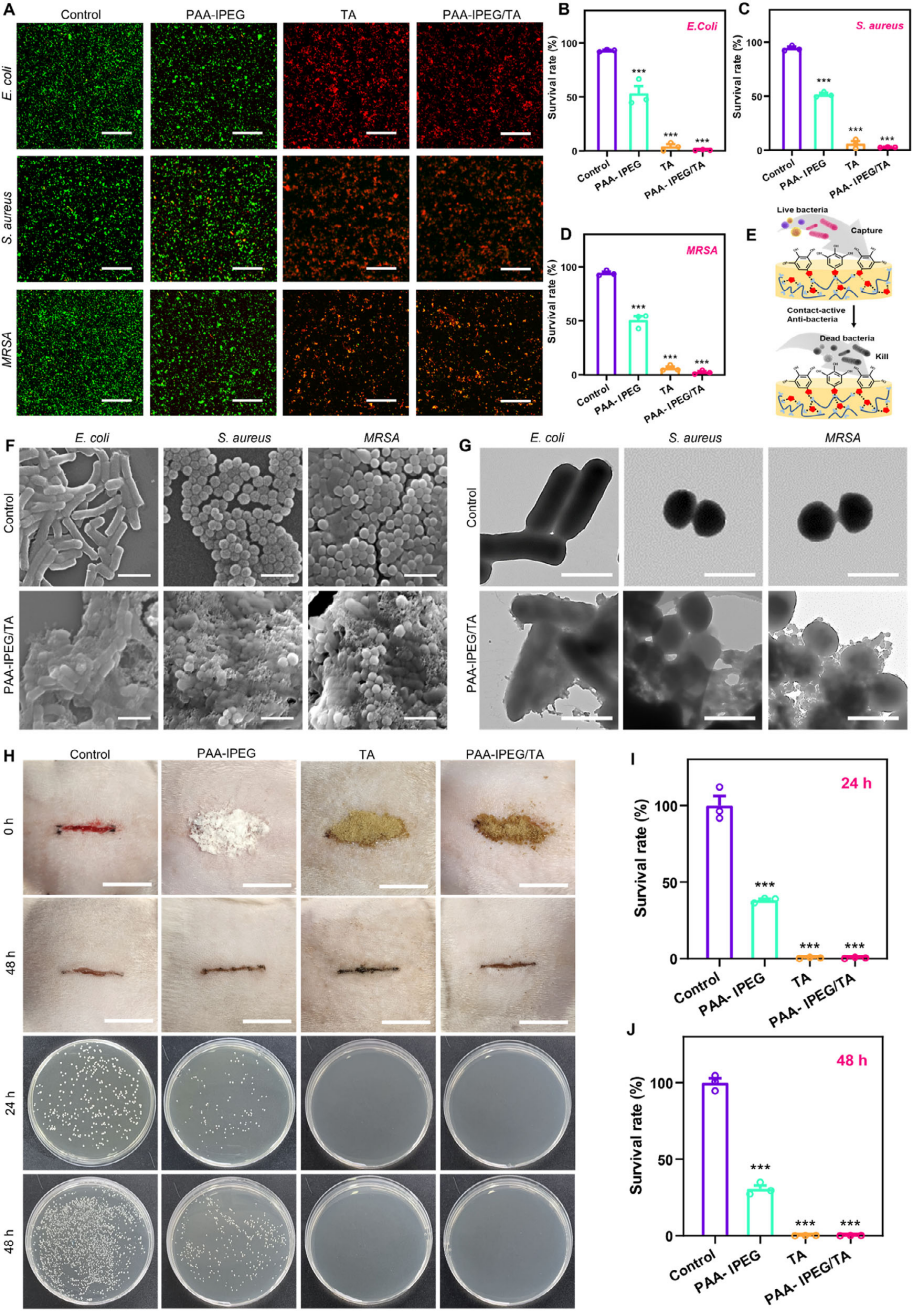



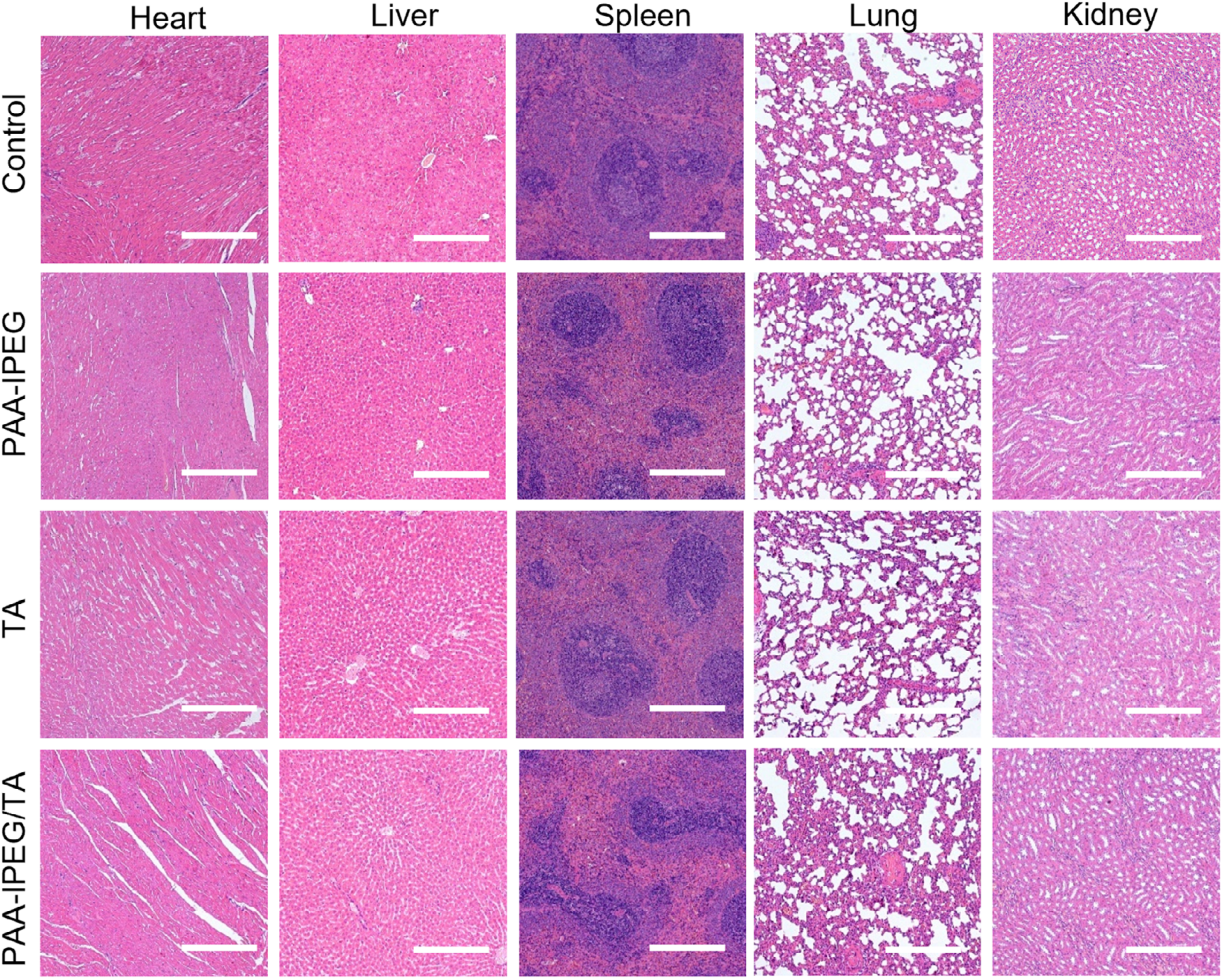


We confirm that this error does not affect any of the results or conclusions of the article. We apologize for this error.

## Supporting information




**Supporting File**: advs76448‐sup‐0001‐SuppMat.docx.

